# A CSI-Based Human Activity Recognition Using Deep Learning

**DOI:** 10.3390/s21217225

**Published:** 2021-10-30

**Authors:** Parisa Fard Moshiri, Reza Shahbazian, Mohammad Nabati, Seyed Ali Ghorashi

**Affiliations:** 1 Cognitive Telecommunication Research Group, Department of Electrical Engineering, Shahid Beheshti University G. C., Tehran 1983969411, Iran; p.fardmoshiri@mail.sbu.ac.ir (P.F.M.); mo.nabati@mail.sbu.ac.ir (M.N.); 2 Electrical Engineering Research Group, Faculty of Technology and Engineering Research Center, Standard Research Institute, Alborz 31745-139, Iran; r.shahbazian@standard.ac.ir; 3 Department of Computer Science & Digital Technologies, School of Architecture, Computing, and Engineering, University of East London, London E15 4LZ, UK

**Keywords:** activity recognition, Internet of Things, smart house, deep learning, channel state information

## Abstract

The Internet of Things (IoT) has become quite popular due to advancements in Information and Communications technologies and has revolutionized the entire research area in Human Activity Recognition (HAR). For the HAR task, vision-based and sensor-based methods can present better data but at the cost of users’ inconvenience and social constraints such as privacy issues. Due to the ubiquity of WiFi devices, the use of WiFi in intelligent daily activity monitoring for elderly persons has gained popularity in modern healthcare applications. Channel State Information (CSI) as one of the characteristics of WiFi signals, can be utilized to recognize different human activities. We have employed a Raspberry Pi 4 to collect CSI data for seven different human daily activities, and converted CSI data to images and then used these images as inputs of a 2D Convolutional Neural Network (CNN) classifier. Our experiments have shown that the proposed CSI-based HAR outperforms other competitor methods including 1D-CNN, Long Short-Term Memory (LSTM), and Bi-directional LSTM, and achieves an accuracy of around 95% for seven activities.

## 1. Introduction

The Internet of Things (IoT) is a dynamic global information network consisting of internet-connected devices [1]. Due to the recent advancements in communication systems and wireless technology over the last decade, IoT has become a vibrant research field [2]. The concept is straightforward; things or objects are connected to the internet and exchange data or information with each other over the network. Applications of IoT improve the quality of life [3]. As one of the main IoT applications, smart houses allow homeowners to monitor everything, including the health, especially for those with disabilities and elderly people, by exerting Human Activity Recognition (HAR) techniques [4]. Additionally, the joint task of HAR and indoor localization can be exerted in smart house automation [4]. A user’s location can change how the IoT devices respond to identical gesture commands. For instance, users can use the “hand down” signal to reduce the temperature of the air conditioner, but they can also use the same gesture to lower the television in front of them [4]. HAR has emerged as one of the most prominent and influential research topics in several fields, including context awareness [5], fall detection [6], elderly monitoring [7], and age and gender estimation [8].

HAR techniques can be categorized into three groups: vision-based, sensor-based, and WiFi-based [7]. Existing sensor-based and vision-based methods for HAR tasks have achieved acceptable results. However, these methods still have limitations in terms of environmental requirements. Strictly speaking, camera-based recognition algorithms are susceptible to environmental factors such as background, lighting, occlusion, and social constraints such as privacy issues. Additionally, in sensor-based methods, people often object to these sensor modalities because they are bothersome or cumbersome. Although the underlying technology employed in these sensors is frequently inexpensive, IoT-connected versions of these sensors can be significantly more expensive due to added wireless hardware and branding. WiFi devices, which are less expensive and power-efficient than the aforementioned technologies, invariant to light, easier to implement, and have fewer privacy concerns than cameras, have recently attracted much interest in various applications [4].

The purpose of WiFi-based activity recognition is to distinguish the executed actions by analyzing the specific effects of each activity on the surrounding WiFi signals. In other words, the individual’s movement affects the propagated signal from WiFi access points and can be used to recognize activities. WiFi signals can be described by two characteristics: Received Signal Strength (RSS) and Channel State Information (CSI) [4]. RSS is the estimated measure of received signals’ power which has been mainly used in indoor positioning [9]. As RSS is not stable compared with CSI, it cannot properly capture dynamic changes in the signal while the activity is performed [10]. As a more informative specification of WiFi signals for HAR tasks, CSI has drawn more attention than RSS over recent years [10]. CSI can save physical layer information from each sub-carrier of the channel. When a person performs a particular activity between the transmitter and receiver, the reflected wireless signals from the body generate a unique pattern [11]. Furthermore, human body shapes, speed of performing an activity, environmental obstacles, and the path of performing an activity can cause different changes to received CSI signals. For instance, if a person walks in a straight line this activity has a different effect on CSI signal, comparing to the experiment that a person walks around a square path. Many WiFi devices use CSI to assess the quality of their connection internally. The device collects the experimental phase and strength of the signal at each antenna for each channel in the provided spectrum, allowing signal disruptions to be identified. The WiFi-based method takes advantage of the ubiquitous nature of radio frequency transmissions while also potentially allowing for developing a system that takes advantage of the existing WiFi infrastructure in smart houses [4].

Although business applications of HAR are in the beginning stages, many studies in this field introduce the issues that must be addressed before any practical action. One of the main issues is the specific hardware/software combination that is required to extract CSI data. After choosing the proper hardware, the collected CSI data can be further used as inputs of the Deep Learning (DL) algorithms for HAR task. The effects of each activity in characteristics of the collected CSI can be used in different DL algorithms to distinguish activities and finally classify them [11].

Since CSI is a time-series data with temporal dependency, Recurrent Neural Network (RNN) and its subsets have been exerted more than other DL algorithms in the HAR task. Long Short-Term Memory (LSTM) and RNN apply sequential processing to long-term information, meaning that these data pass through all cells in the network before reaching the present cell. RNNs structure cannot perform efficiently when we need to analyze long sequences, resulting in vanishing gradients. The vanishing gradient problem persists even when the switch gates and long memory in the LSTM network are maintained [11]. Furthermore, this module requires a significant amount of memory bandwidth due to the complexity of the sequential path and Multi-Layer Perceptron (MLP) layers in each cell. Despite the LSTMs proficiency for prediction and classification tasks in time series, they are incapable of learning terms with greater than 100 terms [12]. Additionally, LSTMs analyze the sequential data in one direction, meaning that only past CSI data will be considered [11]. Accordingly, they cannot distinguish between two similar activities, such as lie down and sit down, which have the same start position but different final positions.

In real-time activity monitoring, especially for elderly people, each activity’s period and further information are essential. Therefore, we consider two approaches: 2D-CNN and Attention-based Bi-directional LSTM. Unlike RNNs and LSTMs, where long-term data is analyzed sequentially, convolutions analyze the data in parallel. Furthermore, the training time of LSTMs is slightly longer than the CNNs, and as a result, they require a greater memory bandwidth for processing. Less consumed time in training and lower computational complexity, along with mentioned problems, encouraged us to use 2D-CNN. Since 2D-CNN has high potential in image processing, we convert CSI data into RGB images. In order to generate RGB images, we made a pseudocolor plot from CSI matrices. Each element of the matrices is linearly mapped to the RGB colormap. Furthermore, we applied BLSTM on raw CSI data. HAR’s performance can be improved by using attention-based BLSTM, which concentrates on regions of greater relevance and assigns them higher weights to improve performance. The main contributions of this research are as follows:

- We exploit Raspberry Pi for CSI data collection and offer a public CSI dataset for seven different activities including sit down, stand up, lie down, run, walk, fall and bend in an indoor environment using the Nexmon CSI tool [13]. Due to reflections induced by human activity, each subcarrier contains critical information that will increase HAR accuracy. The CSI matrices in our dataset are composed of 52 columns (available data subcarriers) and 600 up to 1100 rows depending on the period of each activity. The results demonstrate that this hardware is capable of providing tolerable data that is comparable to traditional technologies.
- We propose a new concept in improving the precision of HAR by converting CSI data into images using pseudocolor plots and feeding them into 2D-CNN. This method overcomes the mentioned limitations of LSTM and also the training time and computational complexity are less than those of other existing methods. We also exert a BLSTM network with an attention layer to address LSTM problems with future information. The results demonstrate that the conversion idea with 2D-CNN outperforms BLSTM in accuracy and consumed time.
- We also perform a deep evaluation by implementing two other algorithms, including 1D-CNN and LSTM, and compare our results with four different models used for HAR, including Random Forest (RF) [14], Hidden Markov Model (HMM) [14], DenseLSTM [15], ConvLSTM [16] and Fully Connected (FC) network [17]. We analyze the performance of our dataset and proposed DL algorithms.

The rest of this paper is organized as follows: Section 2 reviews HAR studies. In Section 3, we provide a brief explanation about CSI, required information on hardware, software and firmware. Furthermore, four used neural network’s structures are discussed in this section. Additionally, we briefly discuss other datasets and their public accessibility. The main contributions of this research are summarized in Section 4. We discuss the device configuration to collect CSI, image generation from CSI, and feeding the data to the neural networks. In Section 5, measurement setups and experimental results are reported, and finally, conclusions are discussed in Section 6.

## 2. Related Works

HAR techniques can be divided into three groups: vision-based, sensor-based, and WiFi-based. Several image-based methods for HAR have been published in recent years, using datasets such as RGB (red, green, and blue [18]), depth [19], and skeleton images [20]. The RGB dataset may not be qualified and robust enough in this method when the video contains considerable sudden camera movements and cluttered background. To this end, Anitha et al. [21] propose a shot boundary detection method. In their proposed method, the features and edges of videos are extracted. The features were then extracted as images and subsequently merged with the video feature and fed into the classifier. The Kernel Principal Component Analysis (KCPA) technique is applied to locate image features and joint features. The preparation process is thus gradually proficient, making the independent vector analysis increasingly realistic for real-life applications. The human activity videos are classified by K-Nearest Neighbor, obtaining better results than other cutting edge activity methods. While capturing an image or video of an activity in RGB format, it generates many pixel values, making it more difficult to distinguish the subject from the surrounding background and resulting in computational complexity. The aforementioned obstacles and the view dependency, background, and light sensitivity impair RGB video-based HAR performance and persuade researchers to exert depth images and other formats of images or videos to improve HAR performance. Most of the methods introduced for HAR utilizing skeleton datasets are confined in various ways, including feature representation, complexity, and performance [22]. In [22], authors propose a 3D skeleton joint mapping technique that maps the skeleton joints into a spatio-temporal image by joining a line across the same joints in two adjacent frames, which is then used to recognize the person’s activities. The 3D skeleton joint coordinates were mapped along the XY, YZ, and ZX planes to address the view dependency problem. They exploit transfer learning models including MobileNetV2, DenseNet121, and ResNet18 to extract features from images [22].

In sensor-based methods, wearable sensors capture activities, causing inconvenience and long-time monitoring unavailability [23]. In the past decade, smartphones have become more powerful with many built-in sensors, including the accelerator, gyroscope. The main impediments in using smartphones for HAR tasks are their higher noise ratio than wearable sensors and fast battery drain [23]. Several researchers have exerted Radio Frequency Identification (RFID) tags to recognize human activities [24]. Authors in [24] present a framework for HAR and activity prediction by using RFID tags. They utilize RFID tags to detect a high-level activity and object usage. Additionally, they employ weighted usage data and gain activity logs. Since human activities are time series data and the next activity is related to the current activity and previous ones, they use LSTM to predict activities with an accuracy of 78.3%. Although RFID tags are cheaper, RFID-based systems cannot achieve high accuracy in crowded environments. Additionally, as mentioned above, vision-based HAR needs cameras installation in the environment, which highly depends on the light source’s consistency and is unable to pass through physical obstacles such as walls. Since indoor spaces such as smart houses, malls, and nursing homes are filled with wireless signals, WiFi-based systems have been exploited more than other approaches in recent years [25].

Due to the growing interest in sensor-less activity detection, the research and industry communities have joined on CSI analytics with the help of neural networks. Common CSI-based applications include a wide range of activity detection scenarios such as WiTraffic [26] to delicate activity recognition systems like Wifinger [27], breathtrack [28]. In [29], authors utilize CSI to sense distinct hand movements. They use predefined windows to monitor activity continuously. This method is time-consuming and yields lower accuracy. To overcome this problem, Wi-Chase [30] does not apply predetermined time windows. Due to detailed correlated information in different subcarriers, Wi-Chase also employs all available subcarriers, unlike Wi-Sleep [31] that uses only a subset of them. The extracted features were trained using machine learning algorithms, including KNN and Support Vector Machine (SVM) [30]. Although different WiFi-based HAR systems have been proposed, one of the major challenges has not been addressed properly. That is, WiFi signal changes are due to the various movement speeds and body types of people. Human activity is made up of many limb movements, such as lifting an arm or leg. The speed and scale of activity can naturally alter according to the scenario or period. Furthermore, physical traits such as body form and height are unique for each person. Therefore, human activity patterns can vary greatly amongst people. To address this problem, a WiFi-based HAR proposed in [15] incorporates synthesized activity data that reduces the influence of activity inconsistency such as varied motion speed. They collect CSI for 10 different activities including make phone calls, jumps, check wristwatch, lie down, walk, play guitar, fast walk, play piano, run, play basketball with Atheros AR9590 WiFi chipset. The combination of CSI spectrogram of overall subcarriers is fed into the network as image inputs. Then, four Dense layers are used to extract spatial features of activities. These features are entered to a convolutional layer. Then, a BLSTM is used to extract tempo features and a linear layer is applied to predict the activities. Three data synthesis methods are combined with eight types of transformation methods, including dropout, Gaussian noise, time-stretching, spectrum shifting, spectrum scaling, frequency filtering, sample mixture, and principal component coefficient. Dense LSTM with consistent accuracy of 90% is applied to efficiently optimize the system for the small-size dataset while keeping the model compact to minimize overfitting.

For multi-class classification based on extracted features such as HAR, a variety of machine learning algorithms such as RF, SVM, and HMM and also DL algorithms such as CNN, RNN, and LSTM can be applied. In [14], they apply RF, HMM, LSTM on their public dataset which have been collected with NIC 5300 with three antennas for six different activities including sit down, stand up, fall, walk, run, and bed. A 90-dimensional vector of CSI amplitude (3 antennas and 30 subcarriers) has been used as the input feature vector. They apply the PCA on the CSI amplitude for denoising, and Short-Time Fourier Transform (STFT) for feature extraction. First, they use RF with 100 trees for classification, which has unacceptable accuracy for bed, sit down and stand up activities. They also apply HMM on the extracted features obtained by STFT and DWT techniques. The accuracy is improved compared to RF, but with higher training time. Although HMM has obtained good results for walk and run activities, it cannot distinguish between stand up, sit down, and bed activities. They also apply LSTM on activities [14]. The LSTM extracts the features automatically and directly from raw CSI without any pre-processing. In other words in contrast to other methods, the LSTM approach does not need PCA and STFT, but it has more training time [14]. The accuracy of LSTM is reported over 75% for all activities in [15].

Since the static objects in an environment can also affect wireless signals and, respectively, HAR model, authors in [17] propose a deep neural network as baseline classifier based on the features for four simple activities, including standing up, sitting down, pushing and picking, performed in two different complex environments. More precisely, they propose a network with shared weight to make a similarity network for two different complex environments. They used one transmit antenna and two receive antennas and make four grayscale images from CSI amplitude and phase. In feature extraction stage, Gabor filter is applied on grayscale images to extract features. Gabor filter extracts spatial information of an image by convoluting the transformed image with a filter at specific wavelength λ and orientation θ [17]. For each gray-scale image, the final output is 5 (the number of λ) × 8 (number of θ) × 2 (mean and standard deviation) = 80, and a vector of dimensions 320 = 4 (number of grayscale images) × 80 are fed into the neural network as the input. They exert three FC hidden layers as the baseline network and two identical branches that share the same weight values as the similarity network. A pair of two random data are selected and fed into the two identical networks simultaneously and each one of them enters the fully connected network. If the two data belonged to the same category of activity, they are labeled as “similar”, otherwise “non-similar”. Their model obtains an accuracy of around 84% overall for the two different environment scenarios.

One of the main issues in Wifi-based HAR is the specific hardware/software combination for CSI data collection. In other words, Linux 802.11n CSI Tool is limited to older linux kernels versions and the required hardware cannot be found easily in market. Following the release of Nexmon CSI Tool [13], it is now possible to extract CSI from a BCM43455C0 wireless chipset, which is used in the Raspberry Pi 3B+ and 4B. As this is a recent release, Ref. [16] examines the performance of the Raspberry Pi 4 in CSI-based HAR. They collect CSI signals for different activities performed in normal life as listed: stand up, sit down, go-to-bed, cook, washing-dishes, brush-teeth, drink, pet a cat, sleeping, walk. They do not apply any denoising filter, as their results are acceptable comparing to other available datasets and also additional filtering may affect important information in data. They pack CSI vectors, collected by Raspberry Pi 4, into windows to train their classification model. As LSTMs and their extensions have been well-suited in HAR task, they use a deep convolutional variant of the LSTM model. They apply two 1D-convolutional layers along with four BLSTM, which have more training time and computational complexity. Their model achieves 92% accuracy which demonstrated the Raspberry Pi 4 capabilities for HAR in smart houses and it can be superseded the Linux 802.11 CSI Tool.

## 3. System Model

### 3.1. Preliminary

Transmitting a signal from the transmitter to the receiver, it is deflected, reflected, and scattered when it comes into contact with obstacles and objects. This results in multipath overlaid signals at the receiver when the signal encounters obstacles and objects [7]. Fine-grained CSI can be used to characterize this procedure. The Orthogonal Frequency-Division Multiplexing (OFDM) modulation is utilized in IEEE 802.11, and it distributes the available bandwidth across several orthogonal subcarriers [14]. Due to the limited bandwidth available, the fading that each subcarrier experiences are represented as flat fading [31]. Therefore, the small-scale fading aspect of the channel can be minimized by employing OFDM techniques. Narrow-band fading per subcarrier causes a considerable variation in the measured channel dynamics. The greatest advantage of employing CSI is that it can catch changes occurring at a single frequency and avoid averaging out changes across all WiFi bandwidth, unlike RSS.

Several subcarriers can be present in the physical link between each pair of transmitter and receiver antennas. As each subcarrier might serve many data streams, the CSI obtained from each subcarrier will be unique [14]. CSI can be represented as a channel matrix for *t* transmit and *r* receiving antennas, a given packet transmission *n*:

(1) $$CSI_{n}=\left(\begin{array}{ccc} H_{1,1} & \cdots & H_{1,r}\\ \vdots & \ddots & \vdots \\ H_{t,1} & \cdots & H_{t,r} \end{array}\right)$$

*H_t,r_* represents a vector that includes complex pairs for each subcarrier. Depending on the hardware we use and channel bandwidth, the number of available subcarriers is different [16]. Raspberry Pi 4 and Tp-link archer c20 paired over 5 GHz in 20 MHz bandwidth can access 56 data subcarriers. *H_t,r_* can be expressed as below for *m* data subcarrier in which *h_m_* is a complex number, containing both amplitude and phase of the CSI:

(2) $$H_{t,r}=\left[h_{t,r,1},\ldots ,h_{t,r,m}\right]$$

### 3.2. Hardware and Firmware

To the best of our knowledge, the specialized hardware/software combinations that is required to extract CSI data, are intel 5300 WiFi Network Interface Card (NIC) (Linux 802.11n CSI Tool) [32], Atheros AR9580, AR9590, AR9344, and QCA9558 (Atheros CSI tool) [33], Raspberry Pi (Nexmon CSI Tool) [13]. The intel 5300 NIC has been used for CSI collection since 2011 [32]. Although many researchers used 5300 NIC, such as [14], this hardware configuration has become less important over time since most laptops with this wireless card are not currently available in the market and third-party tools are required to collect CSI. More precisely, some type of Mini PCIe to PCI-Express Adapter with three antennas is required. Atheros CSI tool, as another 802.11n open-source experimental tool for CSI collection, allows extractions of physical layer wireless communication information, including CSI, RSS, the received payload packet, the timestamp, the data rate, etc. [33]. The ath9k open-source kernel driver supports Atheros 802.11n PCI or PCI-E chips; thus, this tool supports any sort of Atheros 802.11n WiFi chipsets. This tool was released in 2015 and there is more hardware with built-in Atheros 802.11n PCI or PCI-E chips rather than 5300 intel NIC, but more expensive.

The release of Nexmon CSI Tool [13] has enabled CSI extraction from Raspberry Pi 3B+ and 4B, Google Nexus 5, and some routers. One of the Nexmon tool benefits is that it permits several transmit-receive antenna configurations (up to 4 × 4 MIMO). Additionally, it includes customizable CSI collection filters that can extract relevant CSI from selected transmitters and the complete CSI data does not need to be suppressed. Although the Raspberry Pi utilizes a single transmit/receive antenna pairing, its price and prospective capabilities make it a suitable tool in WiFi-based healthcare monitoring in smart houses. Nexmon [13] provided a configuration option to assign a different interface to only the monitored frames after being configured on the host for monitoring on Raspberry Pi. This tool can use up to 80 MHz bandwidth and 242 subcarriers. There are three types of subcarriers in OFDM technology, including null subcarriers, pilot subcarriers, and data subcarriers. Null subcarriers (also called zero) are the unused subcarriers mainly employed as a guard against interference from adjacent channels. The pilot subcarriers do not convey modulated data; nevertheless, they are utilized for channel measurements and synchronization between the transmitter and receiver. Furthermore, pilot subcarriers broadcast using a predetermined data sequence and demonstrate an overhead for the channel. The remaining subcarriers from total subcarriers are called data subcarriers. These subcarriers will exploit the same modulation format as 802.11ac [34]. As mentioned in Table 1, we may have different numbers of subcarriers depending on the PHY standard and bandwidth.

### 3.3. Neural Network

Once an activity is performed between transmitter and receiver, it will affect CSI characteristics. When a person performs a particular activity, the received CSI signals generates a unique pattern [7]. Recently, DL algorithms have been widely used to automatically learn features from the effects of activities on CSI. While having many layers in these algorithms offers improved classification skills, overfitting and performance deterioration become significant when implementing the neural network on a limited amount of dataset. Using traditional strategies such as weight decay, small batch size, and learning rate might not be enough to help avoid this problem. Accordingly, all of the pre-existed WiFi-based systems, such as those in Section 2, would require the implementation of dedicated numbers of particular neural layers to provide the desired performance. In this research, we present custom deep learning models that is best suited for situations with a small size dataset and has less computational complexity and consumed time compared to other methods.

#### 3.3.1. CNN

CNN is a feed-forward neural network that excavates features from data with convolution operations. It contains several layers, including Convolution, Pooling, Dense and Flatten. This classification network requires less pre-processing rather than other classification techniques. Additionally, CNN can learn required filters or characteristics without the assistance of the user. CNNs use filters (also known as the kernel, feature detector) to extract features which are performed using the convolution function [35]. The initial Convolution Layer (ConvLayer) is designed to handle lower-level features, such as edges and color. When we employ several ConvLayers in the network topology, the network can achieve high recognition accuracy since it can also capture high-level features.

**Figure 1:**
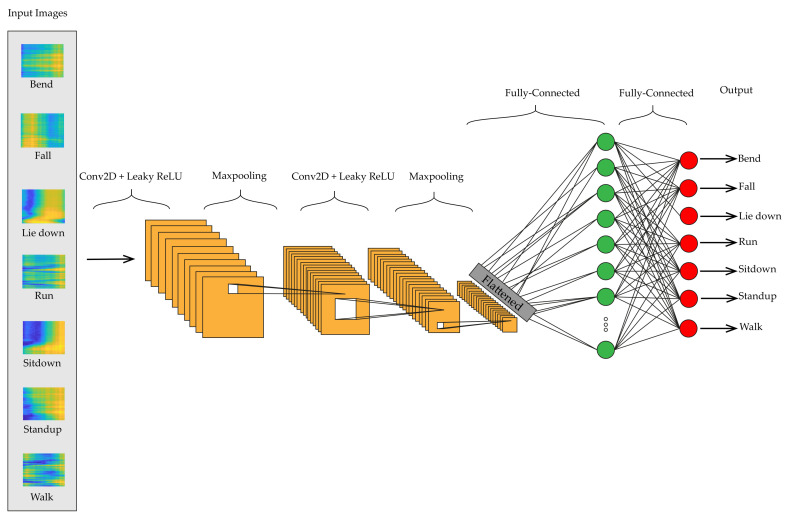
2D-CNN structure used in this paper.

After each two 2D-ConLayer, we use the LeakyReLU activation function, an upgraded variant of ReLU (Rectified Linear Unit). According to the gradient in the negative direction, every value of inputs less than zero causes the gradient to be zero. Therefore, the neurons located in that region are deactivated and may suffer from the dying ReLU problem. In order to address this problem, instead of claiming that negative inputs values should be considered zero, a small linear component of S is defined. LeakyReLU can be formulated as f(S) = max (0.01 × S,S), meaning that if the input is positive, the function returns S and if the input is negative, it returns 0.01 × S. This minor alteration causes a non-zero gradient for negative values; thus, we would not find any dead neurons in that location. Since the feature map output of ConvLayer specifies the specific position of features in the input, a slight movement in the location of the feature in the input data will create a significant difference in the feature map. To address this problem, we use the down-sampling strategy. A better and more widespread strategy is to utilize a pooling layer. After feature detection in ConvLayer, the Max pooling layer is applied to down-sampled feature maps and helps in extracting low-level features. After the first ConvLayer with Leaky ReLU activation function and max pooling, Batch Normalization (B.N.) is applied to stabilize the network during training and speed up training. B.N. makes the variable mean and standard deviation estimations more stable across mini-batches and, respectively, closer to 0 and 1. Dropout layers are applied between convolutional layers, decreasing overfitting while improving the network’s generalization capability. The pooled features (the max pooling’s output) should be flattened. Flattening involves concatenating the feature map matrix to create a single-column matrix. This matrix is passed through a dense layer where we get our predicted classes. The proposed 2D-CNN structure is depicted in Figure 1.

**Figure 2:**
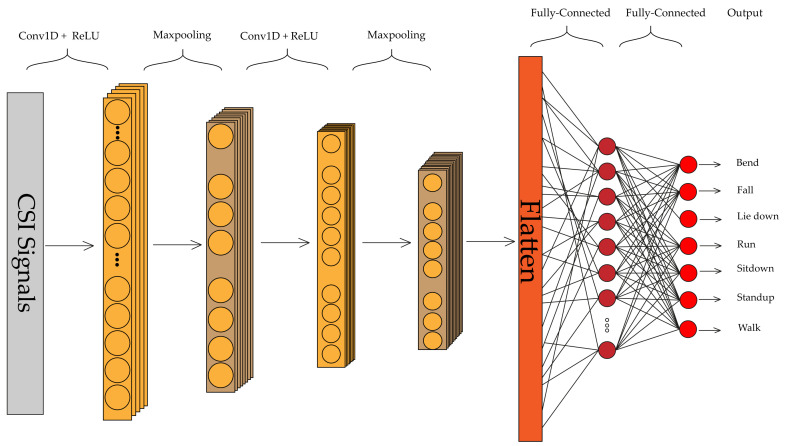
1D-CNN structure used in this paper.

In addition to 2D-CNN exerted on converted RGB images, we also apply 1D-CNN to CSI data as depicted in Figure 2, which will convolve with moving along one dimension. Whether the input is 1D, 2D, or 3D, CNNs all have the same properties and use the same process. The crucial distinction is the dimensionality of the input data and the method in which the filter slides across it. The 1D-CNN has been trained to identify different activities based on sequential observations and map the internal features to different activities. It is particularly good at learning time-series data such as CSI, as it can leverage raw time series data and requires no domain expertise to hand-engineer input features. We use two ConvLayers with ReLU as an activation function. Same as 2D-CNN, after each ConvLayer, we apply max pooling layer, B.N., and dropout.

#### 3.3.2. LSTM

RNN has been successfully applied to sequential modeling applications, such as language understanding [36] and HAR [37]. Nevertheless, when the learning sequence is long, the standard RNN frequently encounters the problem of the gradient vanishing and exploding. In order to address this issue, Hochreiter and Schmidhuber [38] designed a new RNN structure named the LSTM [38]. The LSTM network seeks to overcome gradient vanishing and exploding by utilizing memory cells with a few gates to retain essential information with long-term dependencies. The memory block comprises three gate sets. Each decides the block’s state and produces an output, including forget gate, input gate, and output gate. The information to be eliminated from the unit is determined by the forget gate. The input gate handles which input values cause the memory state to be updated. The output gate determines the output of the block according to the input and the unit memory.

**Figure 3:**
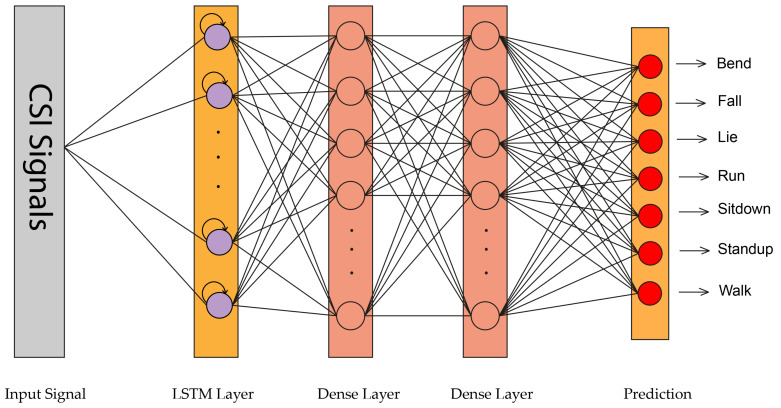
LSTM structure used in this paper.

Since CSI signals are time-series and the LSTM can learn complicated and temporal dynamics, this network has obtained a remarkable performance for CSI-based HAR. In the HAR task, LSTM has two advantages. First, it can extract the features automatically without pre-processing. On top of that, it can hold temporal state information of the activity, resulting in better performance for similar activities such as lie down and sit down comparing to 1D-CNN, RF, and HMM. In this paper, we apply a simple LSTM with one hidden layer and 128 hidden units in which the feature vector is a 52-dimensional vector of CSI amplitudes. The proposed LSTM structure is depicted in Figure 3.

**Figure 4:**
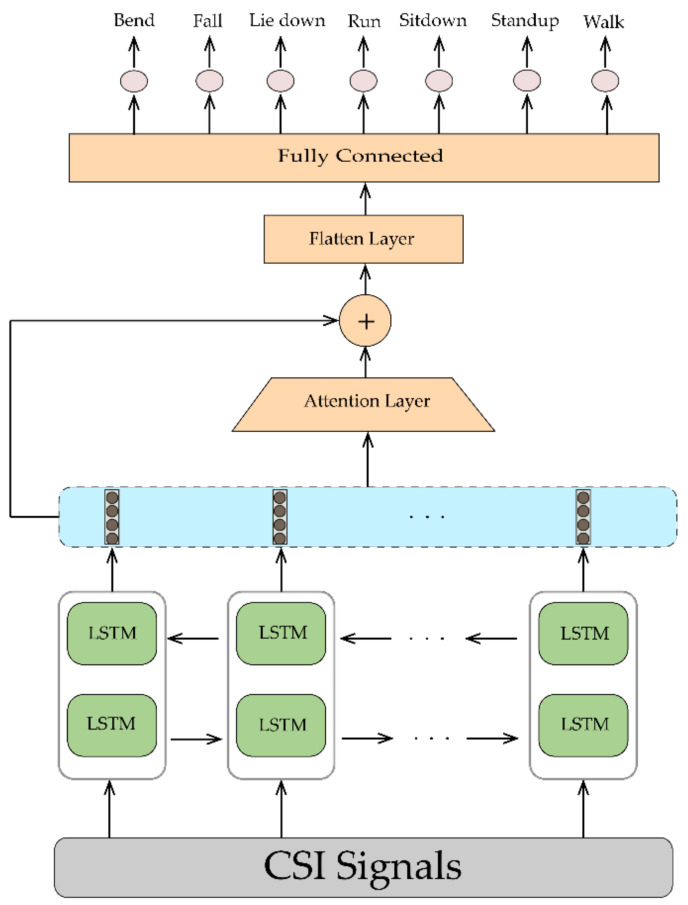
BLSTM structure used in this paper.

The traditional LSTM network only analyze the CSI data in one direction, meaning that the present hidden state only considers the past CSI information. Furthermore, future CSI information is also important for HAR. In this paper, an attention-based BLSTM is utilized to analyze past and future information and overcome long-term dependency. It contains a forward and backward layer for extracting information from the two directions. In other words, it’s a two-layer LSTM sequence processing paradigm: one in which the input moves forward and the other in which the input moves backward. As the name suggests, attention is a technique that can allow input sequences of arbitrary length to pay attention to specified timesteps [11]. The concept is based on the studies about human vision systems, which indicate that humans consistently focus on a certain region of an image while identifying it and then altering their focus over time. It has been found to be effective in image recognition to have the machine focus on the region of interest while concealing the rest of the image at the same time for a recognition task. Due to the sequential features learned by the BLSTM network for WiFi-based HAR known to have high dimensions and feature contributions and time steps may vary from case to case, we seek to exploit the attention model to automatically learn features’ significance and adjust feature weights based on activity recognition performance. In this paper, as depicted in Figure 4, a BLSTM with one attention layer with 400 units is used to learn the relative importance of features and timesteps and more important characteristics are given higher weights to obtain better performance.

The comparison between these four networks and five other networks in HAR researches, i.e., RF [14], HMM [14], DenseLSTM [15], ConvLSTM [16] and FC network [17], are discussed in Section 5. Note that, the proposed networks for our public dataset significantly outperforms other techniques in terms of accuracy, computational and structural complexity, and consumed time.

### 3.4. Human Activity Recognition Datasets

The amount of data we need for the HAR task depends on the complexity of the task and the chosen algorithm, hence there is no specific rule about the number of samples, needed to train a neural network and it is just a process of trial and error. For vision-based HAR task, [39] used 320 samples for 16 activities and [40] used 567 samples for 20 activities. We investigated the quantity of samples utilized in some CSI-based HAR researches. In ConvLSTM [16], they collected CSI data for 11 activities which were performed 100 times in a home environment (1100 samples). In [41], they collected 600 samples from 3 volunteers for 8 activities. In [30], they collected 720 samples of activities (12 volunteers × 20 samples × 3 activities). The authors in [42] collected 50 up to 100 samples for 4 actions (approximately 200 up to 400 samples). In [43], they collected 1400 samples from 25 volunteers. Authors in [44], collected 50 samples for 10 activities (500 samples). Siamak Yousefi et al. [14], as one of the most cited articles in WiFi-based human activity recognition, provided a public dataset for 6 different activities, performed by 6 users for 20 times (720 samples). According to other researches results, we asked 3 volunteers to perform 7 different activities 20 times, resulting in 420 samples. To the best of our knowledge, the WiFi-based researches data accessibility and number of samples are listed in Table 2. Furthermore, it should be mentioned that we plan to increase number of samples and perform activities in different scenarios.

## 4. Proposed Method

**Figure 5:**
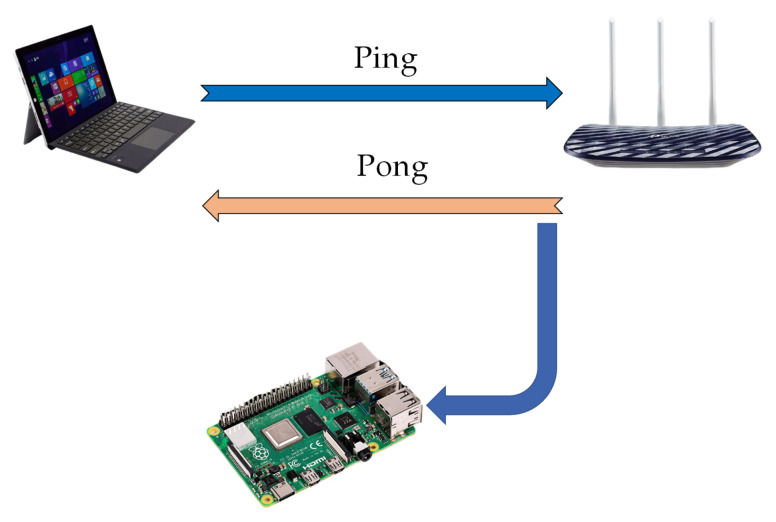
Configuration for CSI collection.

Despite the numerous advantages that accessibility to CSI would provide to users, chip manufacturers continue to treat CSI as a private feature. Only a few devices that are still using the 802.11g and 802.11n technologies are capable of dumping CSI, and they do so with a number of restrictions. Additionally, the Linux 802.11n CSI Tool is only compatible with older Linux kernel versions, which can cause significant inconvenience. In IoT, wireless connectivity is critical for monitoring and control purposes such as HAR. When it comes to experimentation, the Raspberry Pi might be considered a cheap and available WiFi-enabled platform. We employ Nexmon Tool [13] and collect CSI data for seven daily human activities, including walk, run, fall, lie down, sit down, stand up, and bend. We use Raspberry Pi 4 and a Tp-link archer c20 as an Access Point (AP) in 20 MHz bandwidth on channel 36 in IEEE 802.11ac standard. As depicted in Figure 5, we use Personal Computer (PC) for traffic generation by pinging or watching a movie on the internet. The AP will reply with pong packets to the sent pings from the PC. The Pi is in monitor mode and will sniff through this connection and collect CSI for each sent-out pong packet. CSI is saved as a pcap file which can be analyzed in many software including MATLAB. CSI complex numbers are extracted and after removing null and pilot subcarriers, we export activity rows according to the period of each activity which has been detached depending on the video of activity performed by users and stopwatch. Due to reflections induced by human activity, each subcarrier for any given link experiences a variation [11]. Therefore, each subcarrier includes critical information that will increase recognition accuracy. A higher proportion of subcarriers boosts precise feature detection since it provides additional information and boosts identification of challenging features to analyze a subset of subcarriers. The CSI matrices have 52 columns (available data subcarriers) and 600 up to 1100 rows depending on the period of each activity. The dataset is available in GitHub https://github.com/parisafm/CSI-HAR-Dataset (accessed on 27 October 2021).

**Figure 6:**
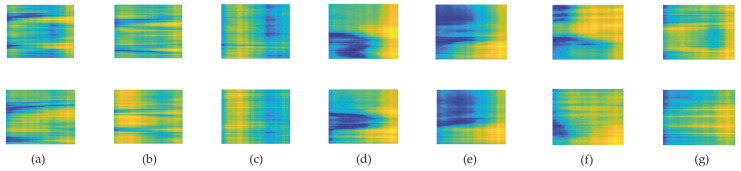
Generated RGB images: (**a**) walk; (**b**) run; (**c**) fall; (**d**) lie down; (**e**) sit down; (**f**) stand up; (**g**) bend.

No data pre-processing is applied on the CSI amplitude since any additional filtering can result in losing important information and affect the system’s performance. If the simulation results or generated images are disappointing, we can use a low pass filter for high-frequency reduction, as mentioned in [16]. In order to make RGB images, the data values must be normalized between 0 and 255 for all activities. We make a pseudocolor plot from matrices representing them as an array of colored faces in the x-y plane. In a pseudocolor plot, cells are arranged in a rectangular array with colors specified by the values in C as normalized CSI input matrices. MATLAB creates this plot by using four points near each corner of C to describe each cell. Each element of C is linearly mapped to the RGB colormap. The generated RGB images are resized to the desired size (64 × 64). Some of these images for each class of activities are depicted in Figure 6. Since the images are not noisy, we do not need to apply denoising filters and additional denoising technique may cause information lost.

These images and CSI data are then fed into neural networks. As CSI signals are typical time-series with temporal dependency, the future information in each step is crucial for HAR, and also LSTMs cannot effectively analyze more than 100 s term, we consider two methods. First, we convert CSI signals to RGB images using pseudocolor plot and feed them into 2D-CNN. By converting CSI to RGB images, the signal pattern for each activity can be seen in one look. Meaning that the pattern changes due to the human movements are depicted in image.

Therefore, in contrast to LSTM that does not have any information about future steps, CNN can analyze the whole signals’ alteration. Additionally, CNN process information parallelly, resulting in faster training than LSTMs with better accuracy. Another method to address LSTMs mentioned problems is to apply BLSTM on CSI data. BLSTM contains a forward and backward layer and can analyze both past and future information by extracting information from the two directions. Since the sequential features learned by the BLSTM network have high dimensions and feature contributions and timesteps may vary for each activity, we exploit the attention layer to learn the relative importance of features. Although BLSTM have high potential to recognize human activities, it needs a greater memory bandwidth for processing and thus it has more training time than the proposed 2D-CNN. Lower consumed time in training and less computational complexity, along with the ability to observe the whole pattern alteration in one look, make the novel image conversion idea and 2D-CNN implementation the best choice over other mentioned methods.

## 5. Evaluation

### 5.1. Measurement Setup

**Figure 7:**
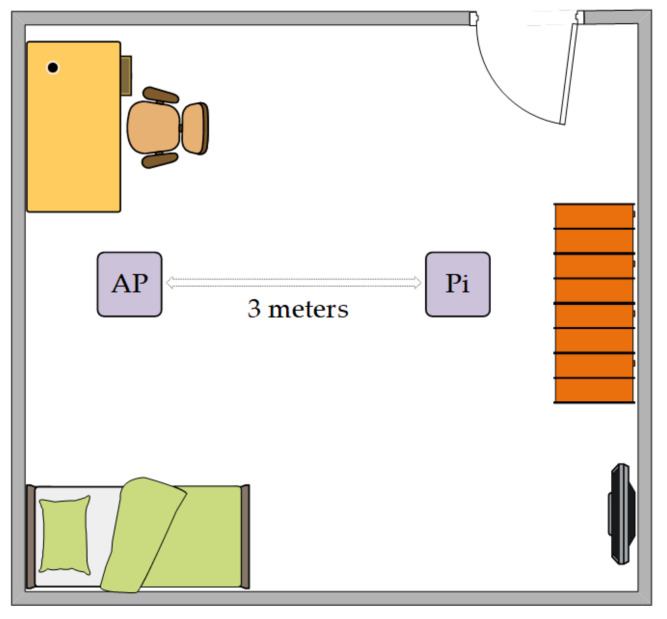
Experimental environment.

Buster lite 4.19.97 raspian and the main branch of nexmon-csi [45] were installed on the Raspberry Pi 4. Nexmon tool was configured as follows: Channel 36, bandwidth 20 MHz, Core 1, NSS mask 1, 4000 samples, 20 s. The AP’s MAC address filter was set to make sure the Raspberry Pi will not connect to another AP on channel 36. The data collection was conducted from another device linked to the Pi over SSH to avoid interference, communicating over another 2.4 GHz network. The AP used is a Tp-link archer c20 wireless router operating a 5 GHz WiFi network on channel 36 at 20 MHz. A PC is paired with the AP to generate traffic by watching a video on the internet or pinging, for which the Pi can capture CSI. We put the Raspberry Pi in monitor mode and with the use of the sniffing method, we were able to collect CSI data. We collect 4000 samples at around 20 s which results in 200 Hz sample rate. Ap and Pi were both 1m above the ground to ensure an unobstructed signal path. They were 3 meters away from each other. The experimental environment is depicted in Figure 7. Each activity performed in the dataset was performed 20 times by three users of different ages. These activities are as listed: fall, stand up, sit down, lie down, run, walk, bend. CSI data were captured in the 20 s, in which an activity has been performed in the middle of this period. More precisely, users remain mostly stable at the start and the end of the capture. As the experiment was managed by the users, the length of time taken for the activity to begin and end may vary slightly, around 3 to 6 s (around 600 to 1100 rows of 4000 total rows). The activity period is extracted according to the video of the activity and stopwatch.

### 5.2. Simulations Results

**Figure 8:**
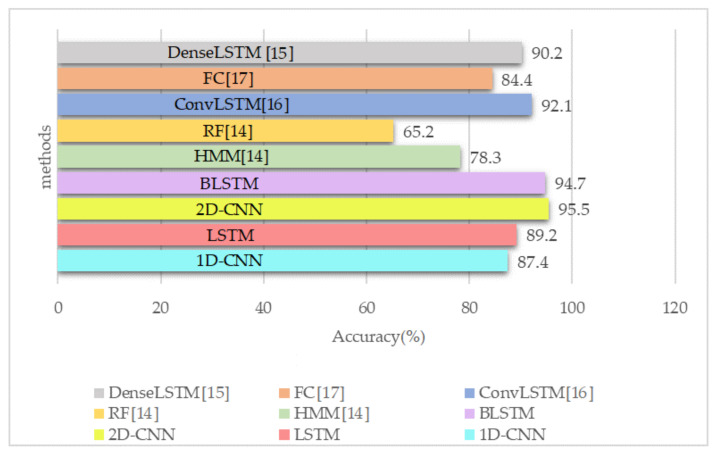
Accuracy of different methods implemented on the dataset.

The proposed deep learning architectures can discover more complex patterns in time series data, compared to hand-crafted features techniques such as RF [14] and HMM [14]. As shown in Figure 8, the ConvLSTM [16] model slightly outperforms the FC network in [17] and DenseLSTM [15]. Our proposed models have achieved better results compared with all of them without any extra data augmentations [15] and complex structure like ConvLSTM [16] and FC [17]. The detailed information about the mentioned methods are available in Section 2. The dataset was split into train and test in a 75% to 25% ratio. We implemented four neural networks on Keras for classification, which has been accelerated by Geforce RTX 2060. The raw CSI amplitude data is a 52-dimensional vector fed into 1D-CNN, LSTM, and attention-based BLSTM. In 1D-CNN model, we have two Conv1D with ReLU as an activation function and after each Conv1D layer, we added a MaxPooling layer. The LSTM network contains one LSTM hidden layer and 128 hidden units. For the BLSTM model, we used one BLSTM layer with 200 hidden nodes and one attention layer with 400 units. The converted RGB images were fed into 2D-CNN with 2xConv2D layer (with Leaky ReLU) and 2xMaxPooling layer (after each Conv2D). The structures of these networks are depicted in Figure 1, Figure 2, Figure 3 and Figure 4.

CNN can detect simple patterns in data, which are subsequently utilized to create more complex patterns within higher layers. 1D-CNN is highly effective when features are derived from fixed-length parts of the dataset and the feature’s position in the section is not crucial, including the analysis of time sequences data (such as gyroscope or accelerometer data or CSI). Since the LSTM network analyzes temporal dependencies in sequential data, it outperforms the 1D-CNN technique. As mentioned in Section 1 and Section 2, LSTMs suffer from vanishing gradient and cannot access next step information. For activities like sit down and lie down which are different at last body movements, it is necessary to have knowledge about next step information. To address these problems, we converted CSI data into RGB images for each activity and used them as inputs for 2D-CNN, thus we can access all the information in past or next steps with one look at images. Additionally, we used BLSTM with attention layer to consider both past and next step information and automatically learn features’ significance to assign higher weights based on HAR performance. The attention-based BLSTM approach and 2D-CNN have achieved the best performance for the recognition of all activities with an accuracy of around 95%. All of these comparisons are depicted in Figure 8.

**Figure 9:**
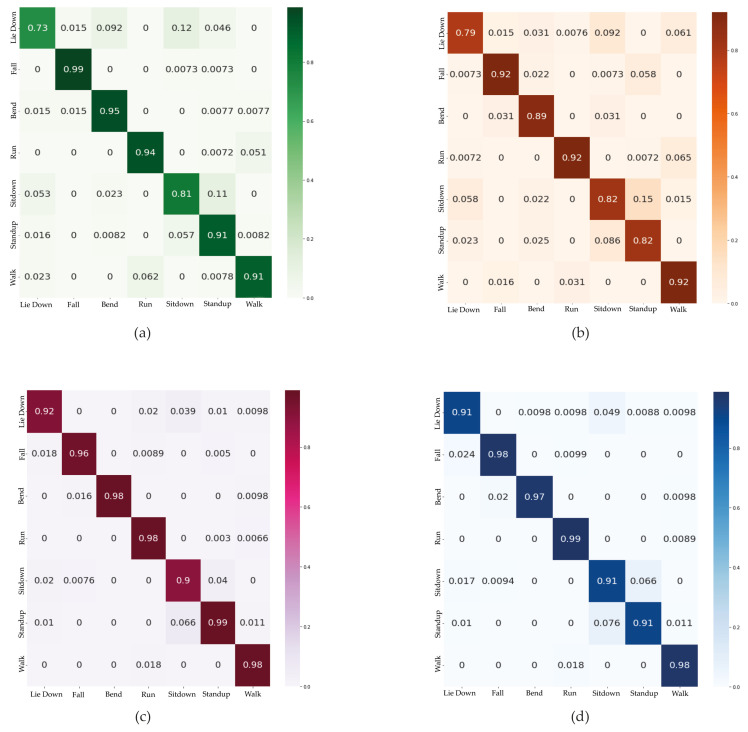
Confusion matrices of proposed methods: (**a**) LSTM; (**b**) 1D-CNN; (**c**) BLSTM; (**d**) 2D-CNN.

Different activities have different CSI values, resulting in different recognition accuracy [7]. We use a confusion matrix (or error matrix) to describe the performance of our proposed classifiers for each activity in which the rows represent anticipated classes and the columns represent actual classes. The activities with more significant body movement, i.e., fall, walk, and run, have higher recognition accuracy (see Figure 9) since they have more influence on CSI characteristics. Furthermore, fall activity is crucial, particularly for elderly healthcare services. Our proposed 2D-CNN and BLSTM network have 98% and 96% accuracy for this activity, making these models efficient in elderly care systems. Another observation is that the action “Lie down” has a recognition accuracy similar to “Sit down” for most methods. The probable explanation is that these activities have a similar impact on CSI values since the start position is the same and the final positions are different. By applying attention-based BLSTM and 2D-CNN, the system is less confused between these two activities. As shown in Figure 9, the model is confused with these two activities around 3% in BLSTM and 2% in 2D-CNN which are acceptable when compared to LSTM with 8% and 1D-CNN with 9% confusion.

Consumed time is another critical performance evaluation indicator representing how much time the model spends training and testing. Table 3 compares the time consumption (milliseconds per step) of six DL approaches: ConvLSTM [16], DenseLSTM [15], LSTM, BLSTM, 1D-CNN, and 2D-CNN. We can observe that proposed 2D-CNN has the shortest time and highest accuracy (Figure 8) compared to the others, making 2D-CNN a better choice compared with BLSTM, ConvLSTM [16], and DenseLSTM [15] in a fraction of the time. More precisely, a long-term input is processed sequentially in LSTMs’ gates, making them not hardware-friendly, as they require greater memory bandwidth to compute parameters, in addition to time-consuming simulations. In contrast, CNN extracts features by utilizing convolution operation, which is easier to compute and faster in training. Furthermore, the CNN accuracy rapidly improved while the BLSTM accuracy slowly improved in a longer training time.

## 6. Conclusions

Due to the ubiquity of WiFi devices, HAR based on wireless signals, including CSI, has witnessed more interest in smart house health monitoring systems. A few CSI datasets for the HAR task collected with 5300 NIC or Atheros PCI chips, are currently available. This paper presented a CSI dataset for indoor HAR using a Raspberry Pi, which is one of the most accessible embedded boards. In this work, we have designed four neural networks to conduct WiFi-based HAR with more than 87% accuracy for our dataset. We used a BLSTM network with an attention layer to address LSTM problems with future information. We also convert CSI data to images using pseudocolor plots and feeding them into 2D-CNN to overcome the mentioned limitations of LSTM. We showed that the idea of CSI conversion to images can obtain high accuracy of 95%, close to BLSTM, which is one of the most successful DL algorithms in time-sequential analysis. Additionally, as CNN processes different features parallelly, it is faster than other methods and less complex in computations. The strong performance of the proposed methods indicates that the data collected by Raspberry Pi can effectively be employed in smart house HAR. The proposed methods can boost elderly health monitoring systems since it meets the requirements for acceptable recognition accuracy and recognition speed for the most commonly performed actions in this task.

Nevertheless, we presented the first version of our public dataset and plan to improve it by investigating different environments and scenarios. In the future, we will study human-to-human interactions and the CSI changes in multiple user-multiple environments scenarios. Since different ages may perform activities differently, according to their physical ability, we collected CSI data from three different ages, including an adult, a middle-aged person, and an elderly person and try to study other ages, including child and teen. Additionally, we will investigate activities with different initial movements, such as standing + walking and running + walking. 

## Figures and Tables

**Table 1:** Subcarrier description for each PHY standard.

PHY Standards	Subcarriers Range	Pilot Subcarriers	Total/Data Subcarriers
802.11a/g	−26 to −1, +1 to +26	−21, −7, +7, +21	52/48
802.11n 802.11ac 20 MHz	−28 to −1, +1 to +28	−21, −7, +7, +21	56/52
802.11n 802.11ac 40 MHz	−58 to −2, +2 to +58	−53, −25, −11, +11, +25, +53	114/108
802.11ac 80 MHz	−122 to −2, +2 to +122	−103, −75, −39, −11, +11, +39, +75, +103	242/234

**Table 2:** Number of samples and data accessibility in different CSI-based HAR researches.

Research	Number of Samples	Public Accessibility
[16]	1100	No
[41]	600	Yes
[30]	720	No
[42]	200–400	No
[43]	1400	No
[44]	500	No
[14]	720	Yes
Our Dataset	420	Yes

**Table 3:** Consumed-time (milliseconds per step) comparison for different models.

Time	1D-CNN	LSTM	2D-CNN	BLSTM	ConvLSTM [16]	DenseLSTM [15]
Train	9	13	15	28	36	60
Test	3	6	7	12	19	41

## Data Availability

The data presented in this study are available in GitHub: https://github.com/parisafm/CSI-HAR-Dataset (accessed on 27 October 2021).
